# N-3 Polyunsaturated Fatty Acids Stimulate Bile Acid Detoxification in Human Cell Models

**DOI:** 10.1155/2018/6031074

**Published:** 2018-04-05

**Authors:** Anna Cieślak, Jocelyn Trottier, Mélanie Verreault, Piotr Milkiewicz, Marie-Claude Vohl, Olivier Barbier

**Affiliations:** ^1^Laboratory of Molecular Pharmacology, CHU de Québec Research Centre and the Faculty of Pharmacy, Laval University, Québec, QC, Canada; ^2^Liver and Internal Medicine, Medical University of Warsaw, Warsaw, Poland; ^3^Translational Medicine Group, Pomeranian Medical University, Szczecin, Poland; ^4^Institute of Nutrition and Functional Foods (INAF) and CHU de Québec Research Centre, Laval University, Québec, QC, Canada

## Abstract

Cholestasis is characterized by the accumulation of toxic bile acids (BAs) in liver cells. The present study aimed to evaluate the effects of n-3 polyunsaturated fatty acids (n-3 PUFAs), such as docosahexaenoic (DHA) and eicosapentaenoic (EPA) acids, on BA homeostasis and toxicity in human cell models. The effects of EPA and/or DHA on the expression of genes involved in the maintenance of BA homeostasis were analyzed in human hepatoma (HepG2) and colon carcinoma (Caco-2) cells, as well as in primary culture of human intestinal (InEpC) and renal (RPTEC) cells. Extracellular BA species were quantified in culture media using LC-MS/MS. BA-induced toxicity was evaluated using caspase-3 and flow cytometry assays. Gene expression analyses of HepG2 cells reveal that n-3 PUFAs reduce the expression of genes involved in BA synthesis* (CYP7A1, CYP27A1)* and uptake* (NTCP)*, while activating genes encoding metabolic enzymes* (SULT2A1)* and excretion transporters* (MRP2, MRP3)*. N-3 PUFAs also generate a less toxic BA pool and prevent the BA-dependent activation of apoptosis in HepG2 cells.* Conclusion*. The present study reveals that n-3 PUFAs stimulate BA detoxification.

## 1. Introduction

Bile acids (BAs) exert an essential role in the control of lipid and cholesterol homeostasis and constitute the major endogenous component of the human bile [1]. Their formation from cholesterol in the liver accounts for 90% of cholesterol catabolism. The hepatic BA synthesizing pathways lead to formation of the primary chenodeoxycholic (CDCA) and cholic (CA) acids which are, in part, conjugated with taurine and glycine to form amidated acids [1]. Conjugated and unconjugated BAs are stored in the gallbladder and secreted in the intestine, where they act as natural detergents to facilitate the absorption of dietary lipids, liposoluble vitamins, and cholesterol [1]. A significant proportion of CDCA and CA can be deconjugated and converted in the respective secondary lithocholic (LCA) and deoxycholic (DCA) acids by resident bacteria from the large intestine [1]. Both primary and secondary acids are reabsorbed and return to the liver via the portal circulation. Back in the liver, LCA and CDCA sustain additional biotransformation into 6*α*-hydroxylated hyodeoxycholic (HDCA) and hyocholic acids (HCA), respectively [1].

Their detergent properties render BAs hepatotoxic at high concentration [2]. Their retention leads to liver dysfunction, as observed when bile secretion is impaired, a situation called cholestasis [3]. BA accumulation in hepatocytes can cause apoptosis [4] or necrosis [5], with unequal contribution of both types of cell damage to liver injury [6]. To overcome these deleterious effects, the liver has developed self-protective mechanisms based on the regulation of BA synthesis, metabolism, and excretion. Accordingly, when BAs accumulate in liver cells, genes controlling their synthesis, such as the cytochrome P450 (CYP)7A1 and 27A1, are repressed [7] leading to an increased formation of the less toxic glyco- and tauro-conjugated BAs [8]. While canalicular BA is exported by the bile salt export pump (BSEP) and multidrug resistance protein (MRP) 2 remains unchanged [9], the elimination of accumulating BAs from the liver is reinforced through the activation of an alternative basolateral transport system involving the organic solute transporter alpha/beta (OST*α*/*β*) and MRP3 and 4 proteins [9, 10]. The liver simultaneously triggers a repression of sodium dependent (Na^+^ Taurocholate cotransporting polypeptide, NTCP) and independent (organic anion transporting polypeptide, OATP) uptake system [10, 11], preventing the influx of BAs from bloodstream. Furthermore, the expression of phase I and phase II BA metabolic enzymes is activated, which facilitates their elimination through the kidney into the urine [12, 13]. This adaptive response also involves regulatory process in important extrahepatic tissues. For example, in the intestine, cholestasis causes an upregulation of the ileal intestinal bile acid binding protein (I-BABP) transporter resulting in an increased absorption of conjugated BAs by enterocytes [10]. In kidney, the apical sodium dependent transporter (ASBT) is downregulated in proximal renal tubuli causing a reduced reabsorption and an increased BA urinary excretion [14].

All the above described mechanisms are regulated by a series of ligand-activated transcription factors, called nuclear receptors (NRs), such as the farnesoid X-receptor (FXR), small heterodimer partner (SHP), pregnane X-receptor (PXR), liver X-receptor (LXR), peroxisome proliferator activated receptor (PPAR*α*), liver receptor homolog- (LRH-) 1, and hepatic nuclear factor (HNF) 4*α* [7, 15]. While being efficient in controlling BA homeostasis under normal situations, these self-protective mechanisms are overtaken under chronic cholestatic conditions, and the accumulation of toxic BAs contributes to the pathogenesis of autoimmune inflammatory diseases such as primary biliary (PBC) and primary sclerosing (PSC) cholangitis [1]. The reduction in BA hepatic levels is therefore an important therapeutic goal of anticholestatic strategies [16].

N-3 polyunsaturated fatty acids (n-3 PUFAs) such as eicosapentaenoic (EPA) and docosahexaenoic (DHA) acids are found in fatty fish and other marine sources and have multiple beneficial health effects on numerous chronic diseases, such as cardiovascular and neurodegenerative diseases or cancers [17]. A series of recent* in vivo* studies, which aimed at evaluating the potential benefits of n-3 PUFAs in the context of cholestasis treatment, revealed controversial observations. Indeed, while Chen and colleagues reported that n-3 PUFAs induce liver fibrosis in bile duct-ligated cholestatic rats [18], a reduction of hepatocellular injury was observed in bile duct-ligated mice administrated with n-3 PUFAs [19]. In clinics, a 12-month open label pilot study revealed that oral DHA allowed a modest but still significant reduction in alkaline phosphatase levels in PSC patients [20]. Since recent investigations also revealed that EPA and/or DHA protect the liver against BA-induced injury [21, 22], we sought to analyze the mechanisms of the hepatoprotective properties of n-3 PUFAs, by (i) evaluating how n-3 PUFAs modulate the BA-related transcriptome in human liver, intestine, and renal cell models; (ii) analyzing whether these compounds affect BA formation and secretion in human hepatoma HepG2 cells; and (iii) determining how EPA and/or DHA affect the BA-dependent activation of hepatic cell apoptosis and necrosis.

## 2. Materials and Methods

### 2.1. Materials

EPA and DHA were obtained from Sigma (St. Louis, MO). Normal and deuterated BAs were purchased from Steraloids Inc. (Newport, RI) and C/D/N Isotopes Inc. (Pointe-Claire, Canada), respectively. Strata X and Synergi RP Hydro columns were from Phenomenex (Torrance, CA). The SYBR Fast PCR Master mix and Enzchek® caspase-3 assay kit were purchased from Thermo (Life Technologies Division, Foster City, CA). Annexin V-FITC and propidium iodine (PI) were from eBioscience (San Diego, CA). Protein assay reagents were obtained from Bio-Rad Laboratories Inc. (Marnes-la-Coquette, France). Fetal bovine serum (FBS) and other cell culture reagents were from Wisent (Québec, QC Canada).

### 2.2. Cell Culture

Human hepatoma HepG2 cells and colon carcinoma Caco-2 cells were obtained from the American Type Culture Collection (Manassas, VA), while RPTEC and InEpC were purchased from Lonza (Walkerville, MD, USA). HepG2 and Caco-2 cells were cultured in Dulbecco's modified Eagle's medium (DMEM) supplemented with 10% FBS, 1% L-glutamine, penicillin/streptomycin, and Nonessential Amino-Acids. RPTEC were cultured in enriched DMEM/HAM-F-12 medium. The smooth Muscle Growth SingleQuot Medium (SmGM), purchased from Lonza, was used for InEpC maintenance. All cells were incubated at 37°C in a humidified CO_2_ incubator except for InEpC, which were maintained, in accordance with the manufacturer's instructions, at 33°C. All experiments were performed with cells at ~80% confluence and serum-free medium.

### 2.3. RNA Isolation, Reverse Transcription, and Quantitative Real-Time PCR (qRT-PCR)

For RNA analyses, 300,000 cells/well were seeded in 12-well plates and cultured in the presence of DMSO (vehicle, 0.1% v/v), EPA, and/or DHA at the indicated conditions. Total RNA was isolated from treated or control cells according to the TriReagent acid : phenol protocol as recommended by the supplier (Molecular Research Center Inc., Cincinnati, OH). The reverse transcription (RT) reaction was carried out using 200 units of Superscript II (Invitrogen, Life Technologies Division) and random hexamer primers (150 ng) with up to 1 *μ*g of total RNA (Invitrogen) at 42°C for 50 min. Real-time PCR quantifications were performed using an ABI ViiA 7 Real-Time Fast PCR system (Thermo-Life Technologies, Carlsbad, CA). For each reaction, the final volume of 10 *μ*L was composed of 5 *μ*L of SyBr Fast PCR Mix, 1 *μ*L of each primer (Supplementary Table ST1), and 3 *μ*L of a RT product diluted from 50 to 1,000 times. Conditions for qRT-PCR were 95°C for 20 sec, 95°C for 30 sec, and annealing temperature (ST1) for 20 sec for 40 cycles. Threshold cycle (Ct) values were analyzed using the comparative Ct (ΔΔCt) method as recommended by the manufacturer (Thermo). The amount of target gene was obtained by normalizing to the endogenous reference Pumilio RNA-Binding Family Member 1 (PUM1) and was expressed relatively to vehicle-treated cells set at 1. For each gene, the amplification efficiency and the accuracies of ΔΔCt of target genes compared with PUM1 were tested using 2 to 5 log of complementary DNA (cDNA) concentrations.

### 2.4. Liquid Chromatography Coupled to Tandem Mass Spectrometry (LC-MS/MS)

BAs were analyzed in culture media from HepG2 cells (300,000 cells/well of 12-well plates) cultured in the presence of 25 and 50 *μ*M of EPA and DHA for 24 H. Nineteen BA species were profiled in 300 *μ*L of culture media using LC-MS/MS as extensively described elsewhere [23]. The chromatographic system consisted of a Prominence liquid chromatography instrument (Shimadzu Scientific Instruments, Columbia, MD, USA) which was coupled to an API4000 instrument equipped with an electrospray ionization source (Applied Biosystems, Concord, Canada).

### 2.5. Fluorescence-Activated Cell Sorting (FACS) and Caspase-3 Assays

Hepatoma HepG2 cells (200,000 cells/well of 12-well plates) were pretreated with DMSO (vehicle, 0.1% v/v) or EPA and/or DHA (10, 25, or 50 *μ*M) for 3, 6, 16, or 24 H and then cultured for up to 24 H in the presence of a BA mixture (CDCA, CA, DCA, and LCA, 100 *μ*M each). For FACS analyses, cells were washed with PBS and resuspended in 100 *μ*L of binding buffer containing 5 *μ*L of Annexin V-FITC and 10 *μ*L of propidium iodine (PI) and incubated in the dark for 15 min. Labeled samples were then analyzed for Annexin V-FITC (525/50 nm) and PI (585/42 nm) using a FACSCanto II instrument (San Jose, CA, USA), at the flow cytometry platform in the CHU de Québec Research Centre (http://services.crchudequebec.ca/services/cytometrie/).

The caspase-3 activity was determined using the EnzChek caspase-3 Assay Kit according to the manufacturer's instructions (Thermo-Life Technologies). Results were analyzed with an Infinite M1000 instrument (Tecan, Austria).

### 2.6. Statistics

All data are presented as mean ± standard deviation (SD). Statistical differences in 3 or more groups were performed using one-way analysis of variance (ANOVA). Statistical differences between 2 groups were analyzed using unpaired two-side* t-*test. Statistical analyses were carried out using SAS statistical software, v9.4 (SAS Institute, Cary, NC, USA).

For BA analyses, the total BA concentration corresponds to the sum of the 19 BA concentrations. The sum of glyco- and tauro-conjugates was calculated by adding the concentrations of conjugated CDCA, CA, DCA, and LCA. The sum of unconjugated BAs also included HDCA and HCA levels.

## 3. Results

### 3.1. N-3 PUFAs Regulate the Expression of Genes Involved in Bile Acid Synthesis, Transport, and Metabolism in Liver Cells

**Figure 1:**
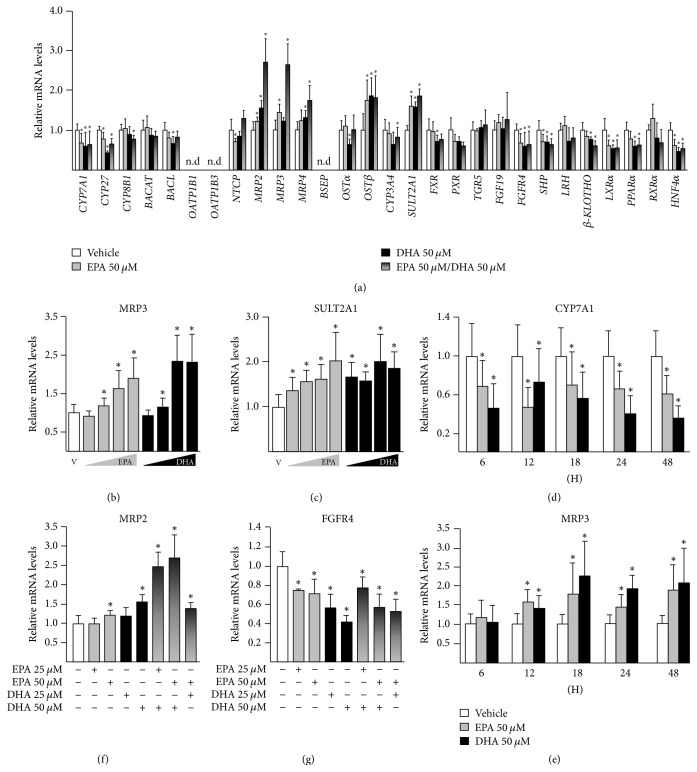
*N-3 polyunsaturated fatty acids EPA and/or DHA modulate the expression of genes involved in bile acid synthesis, transport, and metabolism in human hepatoma HepG2 cells*. Human hepatoma HepG2 cells were treated with DMSO (vehicle, V, 0.1%, v/v) or eicosapentaenoic (EPA) and/or docosahexaenoic (DHA) acids 50 *μ*M (a, d, & e), 5, 15, 25, and 50 *μ*M, (b & c) or 25 and/or 50 *μ*M (f & g) for 24 H (a, b, c, f, & g) or 6, 12, 18, 24, and 48 H (d & e). Total RNA was extracted and analyzed for gene expression using quantitative real-time PCR as detailed in the materials and methods section. Data (mean ± SD) are representative of 2 independent experiments performed in triplicate. Statistically significant differences were analyzed using ANOVA (^*∗*^*p* < 0.05). n.d: not detected.

HepG2 cells were exposed to EPA and/or DHA (50 *μ*M each) for 24 H (Figure 1(a)). Quantitative RT-PCR analyses revealed that EPA and/or DHA significantly downregulate the expression of genes controlling BA synthesis (CYP7A1 and CYP27) and uptake (NTCP), while genes involved in efflux (such as MRP2, MRP3, MRP4, and OST*β*) and metabolism (SULT2A1) were upregulated (Figure 1(a)). Similarly, genes that encode proteins involved in the control of BA signaling were altered by EPA and/or DHA exposure. Indeed, the mRNA levels of NRs, such as FXR, SHP, LXR*α*, HNF4*α*, and PPAR*α*, or membrane receptors (*β*-KLOTHO and fibroblast growth factor receptor [FGFR]4) were downregulated in cells cultured with EPA, DHA, or both compounds (Figure 1(a)).

To further characterize the impact of n-3 PUFAs on the BA-related transcriptome, a series of additional experiments (time-course, dose-response, and cotreatments) were performed (Figures 1(b)–1(d) and supplementary Figures SF1–SF3). These analyses revealed the differential manner in which n-3 PUFAs affect the expression of BA-related genes. For example, some genes (such as* MRP3*) were dose-dependently upregulated by both EPA and DHA, while others were maximally regulated in the presence of the lowest level tested of 1 or 2 compounds (such as* SULT2A1* with DHA) (Figure 1(b)). Other targets, like* CYP7A1, *were regulated as soon as after 6 H exposure to EPA/DHA, while the modulation of others, such as* MRP3 *(Figure 1(c)) and/or* OSTβ* (SF2), required longer exposure. Finally, analyses of cotreatment also evidenced the gene-specific nature of the response to n-3 PUFAs; that is, genes such as* MRP2 *(Figure 1(d)) were additively activated by EPA and DHA, while, for* FGFR4 *(Figure 1(d)),* CYP7A1*, or* CYP27* (SF3), the presence of the 2 n-3 PUFAs did not lead to improved regulatory events, when compared to each omega-3 alone.

To evaluate whether the preparation of PUFAs (i.e., dilution in DMSO) may have an impact on the response of the BA-related transcriptome, an additional experiment was also performed in which HepG2 were exposed to EPA/DHA prepared in DMSO or medium containing 125 *μ*M BSA. As illustrated on SF4, with the exception of CYP27 mRNA levels that were significantly reduced only in BSA-free treated cells, no other significant differences were detected between the 2 formulations.

In summary, these observations indicate that n-3 PUFAs modulate in time-, dose-, and gene-dependent manners the expression of key genes controlling BA homeostasis in HepG2 cells.

### 3.2. N-3 PUFAs Modulate the Secretion of Bile Acids in Hepatoma HepG2 Cells

**Figure 2:**
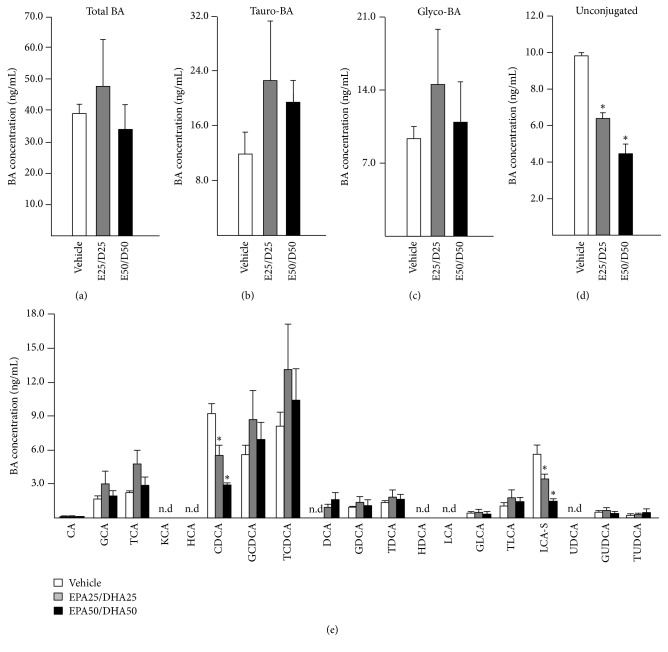
*Eicosapentaenoic and docosahexaenoic acids modulate bile acid secretion by human hepatoma HepG2 cells*. Cells were treated with DMSO (vehicle, 0.1% v/v), 25 or 50 *μ*M eicosapentaenoic (EPA, E), and/or docosahexaenoic (DHA, D) acids for 24 H. Bile acid levels in culture media were measured using LC-MS/MS as described in the materials and methods section. Total (a), tauro- (b), glyco- (c), and unconjugated (d) BAs were calculated based on the measurements of individual BAs (e). The results (mean ± SD) are representative of two independent experiments. Statistically significant differences were analyzed using one-way analysis of variance (ANOVA) (^*∗*^*p* < 0.05). n.d: not detected. BA: bile acid; CA: cholic acid; CDCA: chenodeoxycholic acid; DCA: deoxycholic acid; G: glyco-; HCA: hyocholic acid; HDCA: hyodeoxycholic acid; LCA: lithocholic acid; UDCA: ursodeoxycholic acid; -S: sulfate; T: tauro-.

In order to fully grasp the consequences of the regulatory events described above, we next sought to evaluate how a 24 H exposure to EPA and DHA (25/25 and 50/50 *μ*M) impacts the secretion of BAs in HepG2 cells (Figure 2).

These analyses revealed that total BAs as well as tauro- and glyco-conjugated forms were not significantly affected by EPA/DHA treatment (Figure 2). By contrast, the sum of unconjugated BAs was dose-dependently reduced (Figure 2(d)). As illustrated in Figure 2(e), this reduction mainly reflected the 40.1 and 68.5% significant decrease of CDCA levels observed in media from HepG2 cells cultured in the presence of 25 and 50 *μ*M EPA/DHA, respectively. With the exception of LCA-S, which was also significantly reduced by the EPA/DHA treatment in a dose-dependent manner, concentrations of the other 17 BA species analyzed were not significantly altered (Figure 2(e)).

### 3.3. N-3 PUFAs Exhibit Antiapoptotic but Pronecrotic Effects in HepG2 Cells

**Figure 3:**
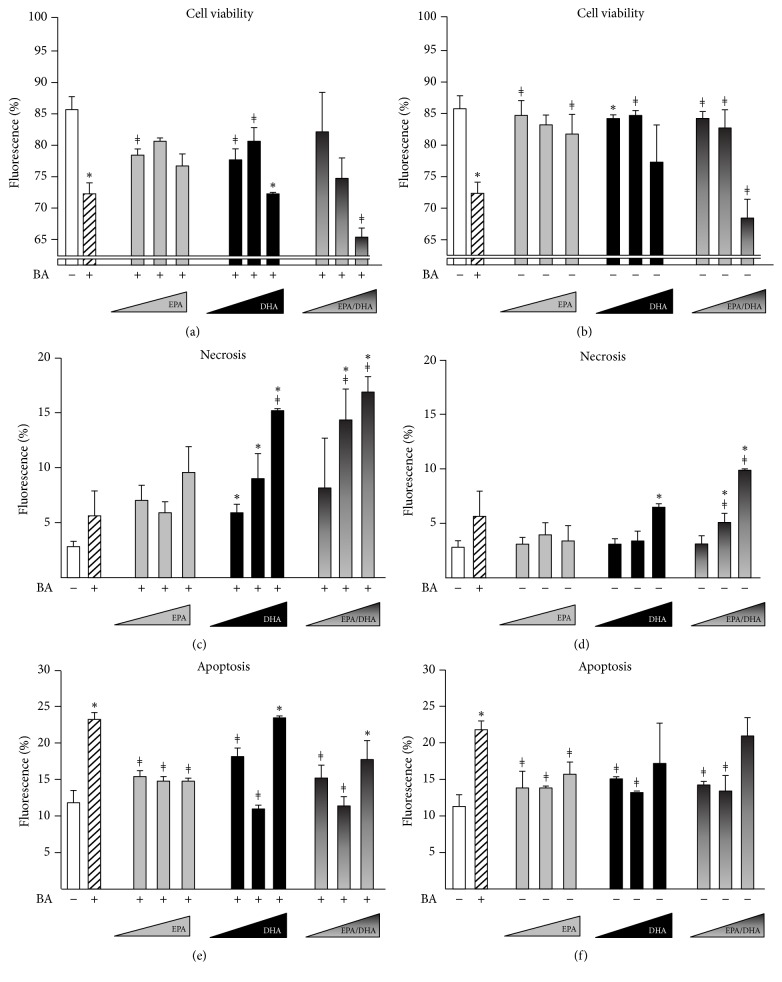
*The n-3 polyunsaturated fatty acids EPA and DHA protect human hepatoma HepG2 cells against the proapoptotic effects of bile acids but worsen their pronecrotic consequences*. Human hepatoma HepG2 cells were cultured in the presence of DMSO (vehicle, 0.1% v/v), eicosapentaenoic (EPA), and/or docosahexaenoic (DHA) acids (10, 25, or 50 *μ*M) for 16 H and exposed (a, c, and e) or not (b, d, and f) to a bile acid (BA) mixture consisting of CDCA, CA, DCA, and LCA, 100 *μ*M each, for 2 H. Cell viability (a, b), necrosis (c, d), and apoptosis (e, f) were determined using fluorescence-activated cell sorting (FACS) analyses. At least 10,000 cells were analyzed for each sample. The results (mean ± SD) are representative of two independent experiments. Statistically significant differences were analyzed using one-way analysis of variance (ANOVA) versus vehicle (*∗*) or bile acids (*ǂ*) (*p* < 0.05).

**Figure 4:**
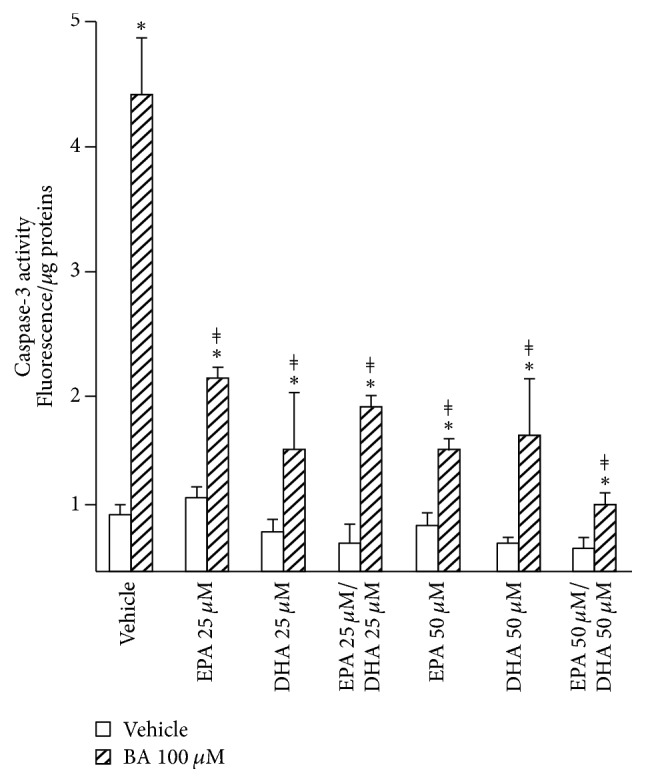
*N-3 polyunsaturated fatty acids reduce the bile acid- (BA-) dependent activation of the caspase 3 activity in HepG2 cells*. HepG2 cells were cultured in the presence of DMSO (vehicle, 0.1% v/v), 25, or 50 *μ*M eicosapentaenoic (EPA) and/or docosahexaenoic (DHA) acids for 24 H and exposed to a bile acid (BA) mixture consisting of CDCA, CA, DCA, and LCA, 100 *μ*M each, for 2 H. The caspase-3 activity was determined as indicated in the materials and methods section. The results (mean ± SD) are representative of two independent experiments performed in triplicates. Statistical differences between two groups were analyzed using unpaired two-side* t-*test versus vehicle (*∗*) or BAs (*ǂ*) (*p* < 0.05).

To further investigate the consequences of n-3 PUFAs on BA toxicity, we next evaluated how EPA and/or DHA affect HepG2 cell viability (Figures 3 and 4).

As illustrated in Figure 3 and as expected from previous studies [24], the 100 *μ*M BA mixture caused a significant reduction in cell viability, as determined by fluorescence-activated cell sorting (FACS) (Figures 3(a) and 3(b)). Interestingly, cells pretreated with low EPA or DHA doses (i.e., ≤25 *μ*M) were more resistant to the BA challenge (Figure 3(a)). However, not only did cells exposed to high DHA doses exhibit a higher sensitivity to toxic BAs (Figure 3(a)), but they were also less viable than control cells in the absence of the BA challenge (Figure 3(b)). The quantification of necrotic cells further revealed that EPA and/or DHA failed to lower the BA pronecrotic effects (Figure 3(c)) and even caused a significant increase of cell necrosis when used alone at high doses (Figure 3(d)). By contrast, when apoptosis was analyzed, EPA and DHA pretreatment did not exhibit statistically significant effects (Figure 3(f)), and, more interestingly, were efficient in preventing the BA-dependent induction of this cell death pathway (Figure 3(e)). Those protective effects against BA-induced apoptosis were further confirmed when measuring the caspase-3 activity (Figures 4 and SF5).

Collectively, these findings indicate that, at low and high dose, n-3 PUFAs protect against the BA-induced apoptosis, while, at elevated concentrations, these fatty acids act themselves as pronecrotic agents and reinforce the BA-induced necrosis in HepG2 cells.

### 3.4. N-3 PUFAs Also Activate Genes Controlling Bile Acid Transport and Metabolism in Human Cell Models for the Intestine and Kidney

To evaluate whether the regulatory events observed in HepG2 cells also occur in human cell models of 2 other tissues involved in BA absorption and/or elimination, namely, the kidney and intestine, additional analyses were performed using human colon carcinoma Caco-2 cells, human intestinal epithelial cells lnEpC, and human renal proximal tubule epithelial cells RPTEC. The optimal response profile was established using dose-response and/or time-course experiments (SF6–SF8).

**Figure 5:**
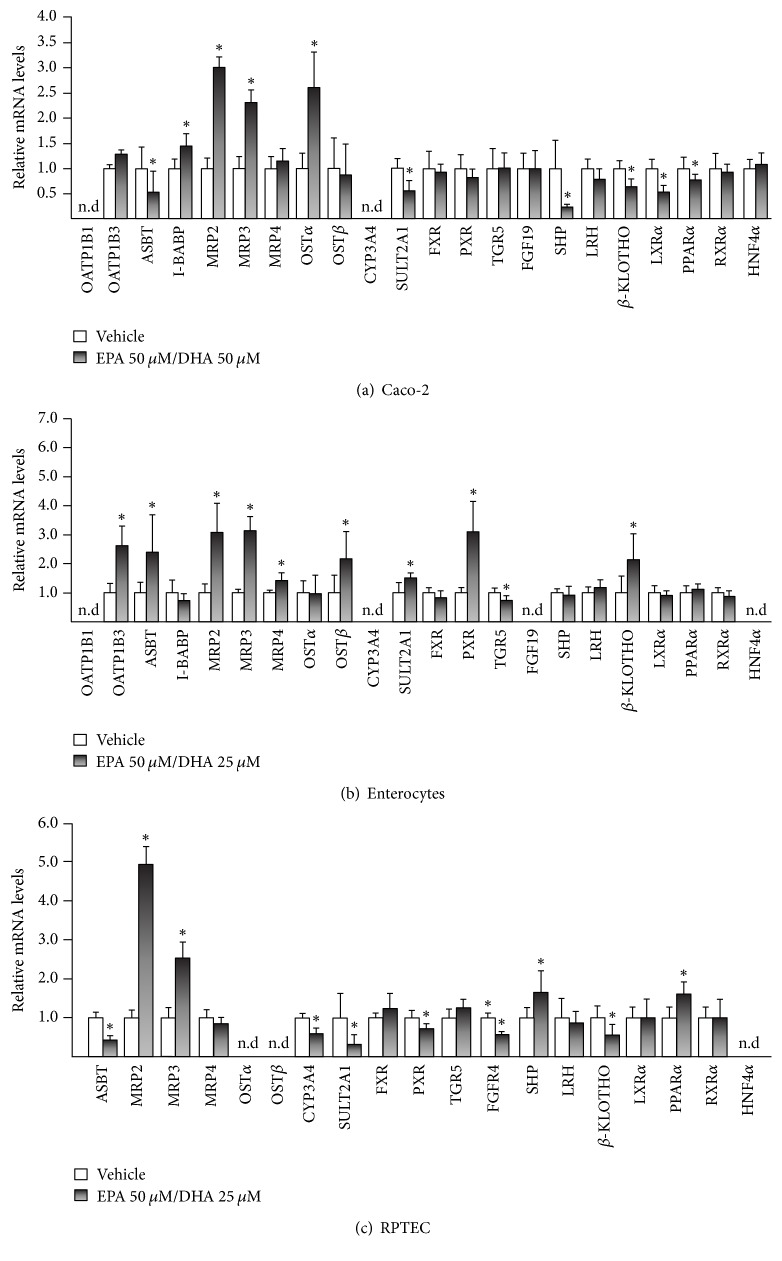
*The n-3 polyunsaturated fatty acids EPA and DHA modulate the expression of genes involved in bile acid synthesis, transport, and metabolism in human intestine and renal cell models*. Human colon carcinoma cells (Caco-2, (a)), enterocytes in primary culture (InEpC, (b)), or renal proximal tubules epithelial cells (RPTEC, (c)) were cultured in the presence of eicosapentaenoic (EPA) (50 *μ*M) and docosahexaenoic (DHA) acids (25 or 50 *μ*M) for 12 (c) or 24 H (a, b). Total RNA was extracted and analyzed for gene expression using quantitative real-time PCR as detailed in Materials and Methods. Data (mean ± SD) are representative of 2 independent experiments performed in triplicate. Statistically significant differences were analyzed using ANOVA (^*∗*^*p* < 0.05). n.d: not detected.

In Caco-2 cells, EPA/DHA (50/50 *μ*M) cotreatment caused a significant modulation in transcript levels of genes involved in BA transport (*ASBT, I-BABP, MRP2/3,* and* OSTα*), metabolism* (SULT2A1)*, and signaling (*SHP, β-KLOTHO,* and* LXRα*) (Figure 5(a)). Even if few genes displayed a cell type-specific response to n-3 PUFAs (for example,* SULT2A1* expression was downregulated in Caco-2 and upregulated in InEpC, Figures 5(a) and 5(b)), a similar response was observed in Caco-2 and InEp cells (Figure 5(b)). Furthermore, n-3 PUFAs also exhibited significant effects on the expression of genes controlling the BA transport, metabolism, and signaling of BAs in RPTEC (Figure 5(c)). Interestingly, in InEpC and RPTEC, 50 *μ*M DHA caused an increased cell mortality and was replaced by a lower dose (25 *μ*M) (Figures 5(b) and 5(c)).

Altogether these observations suggest that, in the intestine and kidney, EPA and DHA also modulate the expression of genes controlling BA detoxification.

## 4. Discussion

**Figure 6:**
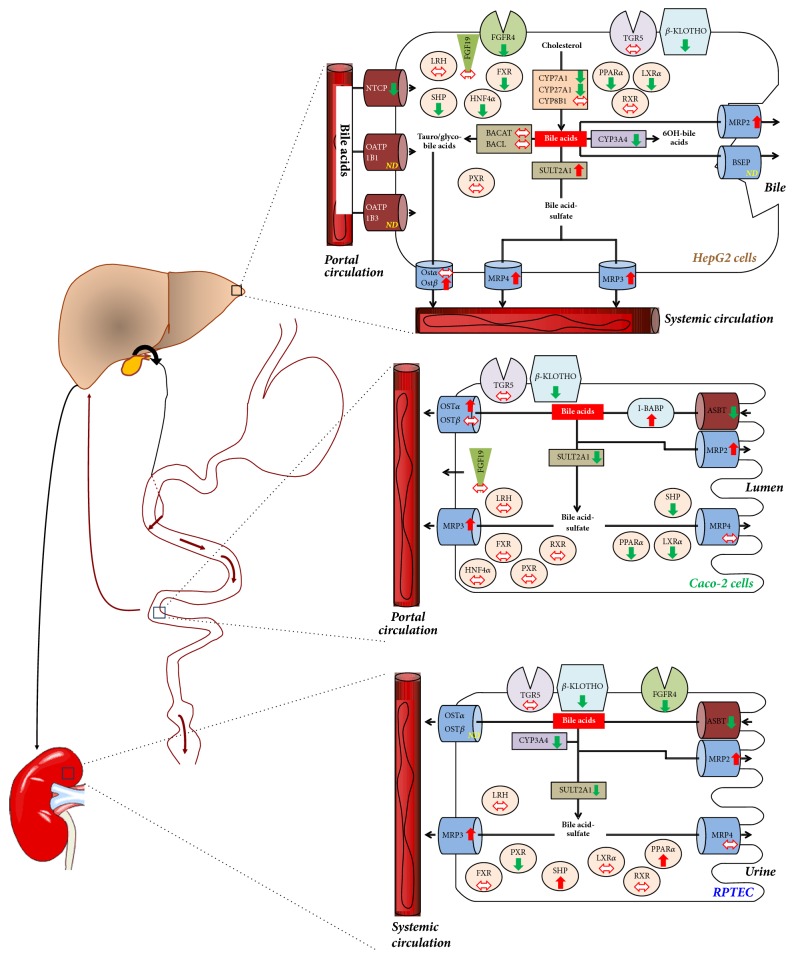
*Coordinated regulation of bile acid synthesis, transport, and metabolism by n-3 polyunsaturated fatty acids in human cell models for the liver, intestine, and kidney*. Results from the present study indicate that n-3 polyunsaturated fatty acids (n-3 PUFAs) such as eicosapentaenoic (EPA) and docosahexaenoic (DHA) acids activate bile acid (BA) detoxification in the liver, intestine, and kidney. In liver (HepG2) cells, EPA and/or DHA dose- and time-dependently reduced mRNA levels of the rate limiting BA synthesizing* CYP7A1* enzyme, while upregulating expression of the sinusoidal* MRP2* and basolateral* OSTα, MRP3,* and* MRP4* BA transporters. The* SULT2A1* enzyme was also significantly upregulated. In Caco-2 and RPTEC, n-3 PUFAs exerted a similar stimulation of the BA detoxification enzymes encoded by* MRP2 *and* MRP3* genes. These regulatory events lead to decreased BA synthesis and increased metabolism and elimination and, so, contribute to reducing systemic accumulation of BAs.

The current study reports a comprehensive analysis of how n-3 PUFAs modulate the BA-related transcriptome in human liver, intestine, and kidney cells (Figure 6) and of how these regulatory events affect BA production and hepatotoxicity.

Results reported herein indicate that EPA and/or DHA activate molecular processes aimed at facilitating the removal of toxic BAs from liver cells. In these cells, n-3 PUFAs reduce transcript levels of the BA synthesizing enzymes* CYP7A1, CYP27,* and* CYP8B1* and of the apical BA importer* NTCP*, while increasing those of the BA exporters* MRP2, MRP3, MRP4,* and* OSTβ* and of the metabolizing* SULT2A1* enzyme (Figure 6). These results are in agreement with previous observations for selected genes such as* NTCP* [25] and* MRP3* [26, 27]. We also observed that genes controlling the transport, metabolism, and signaling of BAs sustain n-3 PUFA-dependent modulation in cellular models of the human intestine and kidney (Figure 6). Even if some genes display an opposite response to the expected one (e.g., the exporter OST*α* and metabolizing enzyme CYP3A4 are downregulated in liver cells), collectively the present observations support the idea that n-3 PUFAs are efficient modulators of genes controlling BA homeostasis in representative models of human tissues exposed to these molecules (Figure 6). Interestingly, the observed reduction in expression of BA synthesizing enzymes was not anticipated, since several studies revealed that n-3 PUFAs fail to either modulate or even upregulate the mRNA expression of the* CYP7A1* [28–30],* CYP27* [29, 30], and/or* CYP8B1* genes [29, 30]. While these discrepancies can reflect differences in experimental settings, our observation that n-3 PUFAs lead to a reduced secretion of the primary BA CDCA in HepG2 cells supports the idea that BA synthesis is effectively decreased in the presence of those fatty acids. A potential species-specific response to n-3 PUFAs could also be considered when interpreting these conflicting observations. Indeed, most of the above cited observations are issued from rodent models [28–30], while the present study was performed using human cell models. Such a possibility is supported by previous studies indicating that genes can be regulated in a species-specific manner [31]. For example, the NR LXR strongly activates the* CYP7A1* gene in rodents [32], but not in humans [31]. Interestingly, LXR is one of the transcription factors identified as a mediator of n-3 PUFAs' effects [33].

The present study also evidences the gene-, PUFA-, and even tissue-selective manner in which EPA and/or DHA regulate the BA-related transcriptome. Actually, these fatty acids modulate the transcription of target genes through various and non-mutually exclusive regulatory mechanisms (i.e., control of transcription factor expression/activity, direct binding to and activation of NRs and membrane receptors, and/or indirect alteration of mRNA stability or gene transcription [34, 35]). Interestingly, most of the EPA/DHA-activated NRs are essential modulators of genes controlling BA synthesis (i.e., PPAR*α*, LXR*α*, and FXR) [33, 36], transport (i.e., PPAR*α*, FXR) [33, 36], and metabolism (i.e., RXR, LXR*α*, PPAR*α*, and FXR) [33, 36]. It can therefore be envisioned that the selective activation of these transcription factors by EPA and/or DHA may play a role in the differential response observed in the present study. Indeed, it has been demonstrated that the relative efficiency of EPA versus DHA to activate NRs participates in their differential biological effects [37, 38].

As discussed above, the BA-related transcriptomic signature of n-3 PUFAs suggests that molecular processes aimed at facilitating the removal of toxic BAs from liver cells are activated. Interestingly, when looking at the herein observed transcriptomic signature of n-3 PUFAs in HepG2 cells, one might get the impression of seeing an FXR signature (repression of NTCP and CYP7A1, induction of MRP2, SULT2A1, and OST*β*) [39], suggesting a potential role for FXR as a mediator of n-3 PUFAs' effects. Actually, n-3 PUFAs activate several nuclear and membrane receptors [40], including FXR [41]. It is therefore plausible that the bile acid sensor contributes to the n-3 PUFA response, and future studies should evaluate such a contribution. The PUFA-dependent reduction in BA toxicity is actually confirmed by the present observation that exposure of HepG2 cells to EPA and DHA causes a significant and dose-dependent reduction of the secretion of unconjugated BAs. Indeed, unconjugated BAs are usually more toxic than their tauro- and glyco-conjugated counterparts [42]. In the same vein, the reduced formation of CDCA leads to a 2- and 3.5-time increase of the CA/CDCA ratio in cells exposed to 25 and 50 *μ*M EPA/DHA, when compared to controls (data not shown). Even if this reduction failed to reach statistical significance, and considering that CA is less toxic than CDCA, this increased ratio is also thought to participate in the reduction of the bile acid pool allowed by n-3 PUFAs [43].

Beyond BA synthesis and secretion, the hepatoprotective effects of EPA and/or DHA are further evidenced by the observation that exposure to low dose of n-3 PUFAs prevents the BA-induced mortality of HepG2 cells. These results indicate that, in promoting the formation of a less toxic BA profile, n-3 PUFAs (at low concentrations) are also efficient in limiting the hepatotoxicity of already formed acids. However, separate analyses of the 2 main pathways involved in cell death (i.e., apoptosis and necrosis) revealed conflicting results. Indeed, while EPA and/or DHA protect against BA-induced apoptosis, they also exhibit dose-dependent pronecrotic effects by themselves and worsen the necrosis induced by BAs. Considering that cytotoxic primary and secondary BAs induce hepatocyte apoptosis at low concentrations (<100 *μ*M) and necrosis in the presence of higher doses [4, 44], these observations suggest that n-3 PUFAs would be more efficient in protecting liver cells against moderated accumulation of BAs as it occurs during chronic cholestasis [8]. Interestingly, similar antiapoptotic effects are thought to participate in the therapeutic benefits observed for ursodeoxycholic acid (UDCA) that is currently used in clinical practice to treat patients with chronic cholestatic liver diseases [45]. Together, these observations further confirm that n-3 PUFAs could be important protective agents against apoptosis, a process participating in the pathogenesis of the chronic cholestatic liver disease, PSC [46, 47]. It is therefore tempting to speculate that the reduction of BA-induced apoptosis participates in the beneficial effects of n-3 PUFAs recently reported in PSC patients [20]. By contrast, in the presence of elevated BA concentrations, as observed under acute cholestatic conditions, such as biliary obstruction [23], n-3 PUFAs may be less favorable. Actually, the presently observed pronecrotic effects can be paralleled to the previous observation that fish fatty acids induce liver fibrosis in bile duct-ligated rats [18]. Indeed, bile duct ligation is representative of a total biliary obstruction, and liver necrosis participates in fibrosis development [18].

## 5. Conclusion

In conclusion, the present study demonstrates that n-3 PUFAs coordinately activate molecular and cellular mechanisms controlling the BA detoxification system in human liver, intestine, and kidney cells. It also evidences that n-3 PUFAs may be protective agents against moderate bile acid accumulation as observed under chronic cholestatic conditions, while it may be worsening for acute cholestasis. Further investigations aimed at identifying the most efficient and safe conditions of treatment (i.e., doses and disease staging) allowing an optimal therapeutic response are however required to fully validate the therapeutic potential of n-3 PUFAs in cholestatic treatment.

## Abbreviations

See supplementary materials.

## Supplementary Materials

Supplementary Materials Supplementary Table 1: primers and conditions used for quantitative real-time PCR experiments. Supplementary Figure 1: dose-dependent and gene-specific modulation of the bile acid-related transcriptome in human hepatoma HepG2 cells treated with EPA and DHA. Supplementary Figure 2: time-dependent and gene-specific modulation of the bile acid-related transcriptome in human HepG2 cells treated with EPA and DHA. Supplementary Figure 3: additive and/or synergistic effects of the EPA+DHA combination on the bile acid-related transcriptome in human hepatoma HepG2 cells. Supplementary Figure 4: preparation of the PUFA solution has minimum impact on the response of BA-related genes in HepG2 cells. Supplementary Figure 5: time-dependent effects of n-3 PUFAs on the bile acid-induced activation of the proapoptotic caspase 3 pathway in human hepatoma HepG2 cells. Supplementary Figure 6: dose-dependent and additive/synergistic effects of EPA and/or DHA on the bile acid-related transcriptome in human colon carcinoma Caco-2 cells. Supplementary Figure 7: time-dependent and gene-specific modulation of the bile acid-related transcriptome in colon carcinoma Caco-2 cells treated with EPA and DHA. Supplementary Figure 8: time-dependent and gene-specific modulation of the bile acid-related transcriptome in RPTEC treated with EPA and DHA.
