# Supplementary material for: N-3 Polyunsaturated Fatty Acids Stimulate Bile Acid Detoxification in Human Cell Models
Source: Can J Gastroenterol Hepatol. 2018 Apr 5;2018:6031074. doi: 10.1155/2018/6031074 (PMC5907406; doi:10.1155/2018/6031074)
Supplement: Supplementary Materials — Supplementary Table 1: primers and conditions used for quantitative real-time PCR experiments. Supplementary Figure 1: dose-dependent and gene-specific modulation of the bile acid-related transcriptome in human hepatoma HepG2 cells treated with EPA and DHA. Supplementary Figure 2: time-dependent and gene-specific modulation of the bile acid-related transcriptome in human HepG2 cells treated with EPA and DHA. Supplementary Figure 3: additive and/or synergistic effects of the EPA+DHA combination on the bile acid-related transcriptome in human hepatoma HepG2 cells. Supplementary Figure 4: preparation of the PUFA solution has minimum impact on the response of BA-related genes in HepG2 cells. Supplementary Figure 5: time-dependent effects of n-3 PUFAs on the bile acid-induced activation of the proapoptotic caspase 3 pathway in human hepatoma HepG2 cells. Supplementary Figure 6: dose-dependent and additive/synergistic effects of EPA and/or DHA on the bile acid-related transcriptome in human colon carcinoma Caco-2 cells. Supplementary Figure 7: time-dependent and gene-specific modulation of the bile acid-related transcriptome in colon carcinoma Caco-2 cells treated with EPA and DHA. Supplementary Figure 8: time-dependent and gene-specific modulation of the bile acid-related transcriptome in RPTEC treated with EPA and DHA. [file 6031074.f1.pdf]

N-3 POLYUNSATURATED FATTY ACIDS STIMULATE BILE ACID DETOXIFICATION IN HUMAN CELL MODELS.

Anna Cieślak, Jocelyn Trottier, Mélanie Verreault, Piotr Milkiewicz, Marie-Claude Vohl and Olivier Barbier

SUPPLEMENTARY MATERIALS

LIST OF ABBREVIATIONS

ASBT,	apical sodium-dependent bile acid transporter
BACAT,	bile acid-Coenzyme A dehydrogenase: amino acid n-acyltransferase
BACL,	bile acid-CoA ligase
BAs,	bile acids
BSEP,	bile salt export pump
CA,	cholic acid
Caco2,	human epithelial colorectal adenocarcinoma cells
CDCA,	chenodeoxycholic acid
CYP,	cytochrome P450
CYP27A1,	sterol 27-hydroxylase
CYP3A4,	cytochrome P450 3A4
CYP7A1,	cholesterol 7α-hydroxylase
CYP8B1,	12α-hydroxylase
DCA,	deoxycholic acid
DHA,	docosahexaenoic acid
DMEM,	Dulbecco’s modified Eagle’s medium
DMEM/HAM-F-12,	Dulbecco's Modified Eagle's Medium and Ham's F-12 Nutrient Mixture
EPA,	eicosapentaenoic acid
FACS,	fluorescence-activated cell sorting
FBS,	fetal bovine serum
FGFR4,	fibroblast growth factor receptor 4
FGF-19,	fibroblast growth factor 19
FXR,	farnesoid X-receptor
GBA,	glyco-conjugated bile acid
HCA,	hyocholic acid

HDCA,	hyodeoxycholic acid
HepG2,	human hepatocellular liver carcinoma cells
HNF4α,	hepatic nuclear factor-4
I-BABP,	intestinale bile acid-binding protein
InEpC,	Intestinal Epithelial Cells
KCA,	ketocholic acid
LCA,	lithocholic acid
LCA-S,	lithocholic acid sulfate
LC-MS/MS,	liquid chromatography-tandem mass spectrometry
LRH-1,	Liver receptor homologue-1
LXRα,	liver X-receptor alpha
MRP,	multi-drug resistance protein
NR,	nuclear receptor
NTCP,	Na^+ Taurocholate cotransporting polypeptide
N-3 PUFAs,	polyunsaturated fatty acids
OATP,	organic anion transporting polypeptide
OSTα/β,	organic solute transpoter alpha/beta
PBC,	primary biliary cholangitis
PI,	propidium iodine
PPARα,	peroxisome proliferator-activated receptors alpha
PSC,	primary sclerosing cholangitis
PUM-1,	pumilio RNA-binding family member 1
PXR,	pregnane X-receptor
qRT-PCR,	quantitative reverse-transcription polymerase chain reaction
RPTEC,	renal proximal tubule epithelial cells

RT,	reverse transcription
RXR,	retinoid X receptor
SHP,	small heterodimer partner
SmGM,	smooth Muscle Growth SingleQuot Medium
SULT,	sulfotransferase
TBA,	tauro-conjugated bile acid
TGR5,	G-protein-coupled bile acid receptor Gpbar1
UDCA,	ursodeoxycholic acid

Supplementary Table 1: Primers and conditions used for quantitative real-time PCR experiments.

Gene	Primers	Annealing Temperature (°C)	RT dilution
ASBT	Sense: 5′-TGACCACATGCTCCACACTG<br>Antisense: 5′-CCCAGAGTCGACCCACATTT	62°C	Caco-2 1/50<br>InEpC 1/25<br>RPTEC 1/50
BACAT	Sense: 5′-CTGCCAACTTTCTCCTGAGACA<br>Antisense: 5′-CCAATCTGTACTCCTTGACATACA	60°C	HepG2 1/200
BACL	Sense: 5′-GTGGAGGGCGTGTTGTCGCA<br>Antisense: 5′-CCGTCGAAAGTCTGGCCGGG	62°C	HepG2 1/200
BSEP	Sense: 5′-GGGCCATTGTACGAGATCCTAA<br>Antisense: 5′-TGCACCGTCTTTTCACTTTCTG	61°C	HepG2 1/50
CYP3A4	Sense: 5′-CCAAGCTATGCTCTTCACCG<br>Antisense: 5′-TCAGGCTCCACTTACGGTGC	65°C	HepG2 1/50<br>RPTEC 1/50
CYP27	Sense: 5′-CGGCAACGGAGCTTAGAGG<br>Antisense: 5′-GGCATAGCCTTGAACGAACAG	60°C	HepG2 1/200
CYP7A1	Sense: 5′-AGAAGCATTGACCCGATGGAT<br>Antisense: 5′-AGCGGTCTTTGAGTTAGAGGA	59°C	HepG2 1/50
CYP8B1	Sense: 5′-GAAGCGCATGAGGACCAAG<br>Antisense: 5′-TTGCATATTGCCCAAAGTCTAGT	59°C	HepG2 1/50
FGF19	Sense: 5′-CGGAGGAAGACTGTGCTTTCG<br>Antisense: 5′-CTCGGATCGGTACACATTGTAG	62°C	HepG2 1/50<br>Caco-2 1/200<br>InEpC 1/25<br>RPTEC 1/50
FGFR4	Sense: 5′-GAGGGGCCGCCTAGAGATT<br>Antisense: 5′-CAGGACGATCATGGAGCCT	62°C	HepG2 1/200<br>Caco-2 1/200<br>InEpC 1/25<br>RPTEC 1/50
FXR	Sense: 5′- GGTGTTTTAACAGAACAAGTGGC<br>Antisense: 5′-ACATTGCTGTATTGCGAGTATGG	60°C	HepG2 1/200<br>Caco-2 1/50<br>InEpC 1/25<br>RPTEC 1/500
HNF4α	Sense: 5′-CGACACGTCCCCATCAGAAG<br>Antisense: 5′-CTCGAGGCACCGTAGTGTTT	60°C	HepG2 1/200<br>Caco-2 1/500<br>InEpC 1/50<br>RPTEC 1/100
IBABP	Sense: 5′-ACCGGCAAGTTCGAGATGG<br>Antisense: 5′-CCTTTTCGATTACATCGCTGGA	60°C	Caco-2 1/50<br>InEpC 1/25
βKLOTHO	Sense: 5′-TTGCCAACGCAAAAGGTCTG<br>Antisense: 5′-GCCAAAGGCAAATCCCAGTG	60°C	HepG2 1/500<br>Caco-2 1/500<br>InEpC 1/50<br>RPTEC 1/100
LRH	Sense: 5′- GAATGCGTGGAGGAAGGAATAA<br>Antisense: 5′-GTCAGAGGGCATAGCTTGGAT	60°C	HepG2 1/200<br>Caco-2 1/500<br>InEpC 1/50<br>RPTEC 1/50
LXRα	Sense: 5′-GCTGCAAGTGGAATTCATCAACC<br>Antisense: 5′-ATATGTGTGCTGCAGCCTCTCCA	64°C	HepG2 1/100<br>Caco-2 1/500<br>InEpC 1/50<br>RPTEC 1/50
**Gene**	**Primers**	**Annealing Temperature**	**RT dilution**

		(°C)	
MRP2	Sense: 5′-CAAACTCTATCTTGCTAAGCAGG<br>Antisense: 5′-TGAGTACAAGGGCCAGCTCTA	59°C	HepG2 1/1000<br>Caco-2 1/1000<br>InEpC 1/50<br>RPTEC 1/50
MRP3	Sense: 5′-CAGAGAAGGTGCAGGTGACA<br>Antisense: 5′-CTAAAGCAGCATAGACGCCC	59°C	HepG2 1/200<br>Caco-2 1/50<br>InEpC 1/100<br>RPTEC 1/50
MRP4	Sense: 5′-GGACAAAGACAACTGGTGTGCC<br>Antisense: 5′-AATGGTTAGCACGGTGCAGTGG	64°C	HepG2 1/200<br>Caco-2 1/1000<br>InEpC 1/50<br>RPTEC 1/500
NTCP	Sense: 5′-TGATATCACTGGTCCTGGTTCTCA<br>Antisense: 5′-GCATGTATTGTGGCCGTTTG	61°C	HepG2 1/50
OATP1B1	Sense: 5′-TGGTCCACCAACAACTGTGGCA<br>Antisense: 5′-AGACAAGCCCAAGTAGACCCTTGAA	60°C	HepG2 1/50
OATP1B3	Sense: 5′-AAGTTGTGCTTTGCGATGCTGAGT<br>Antisense: 5′-GTCAGGCCCTCTAGGAGGTGGG	62°C	HepG2 1/50
OSTα	Sense: 5′-AGATTGCTTGTTCGCCTCC<br>Antisense: 5′-ATTCGTGTCAGCACAGTCATTAG	59°C	HepG2 1/50<br>Caco-2 1/50<br>InEpC 1/50<br>RPTEC 1/50
OSTβ	Sense: 5′-CAGGAGCTGCTGGAAGAGAT<br>Antisense: 5′-GACCATGCTTATAATGACCACCA	59°C	HepG2 1/50<br>Caco-2 1/50<br>InEpC 1/50<br>RPTEC 1/50
PPARα	Sense: 5′- ATATCTCCCTTTTTGTGGCTGCTA<br>Antisense: 5′-TCCGACTCCGTCTTCTTGATGA	60°C	HepG2 1/200<br>Caco-2 1/100<br>InEpC 1/50<br>RPTEC 1/100
PUM1	Sense: 5′-CCGTCGAAAGTCTGGCCGGG<br>Antisense: 5′-CATTAATTACCTGCTGGTCTGAAGGA	62°C	All cells: 1/50
PXR	Sense: 5′-GACAGTGCCAGGCCTGCCGCC<br>Antisense: 5′-CATCTGAGCGTCCATCAGCTCC	62°C	HepG2 1/100<br>Caco-2 1/100<br>InEpC 1/50<br>RPTEC 1/50
RXR	Sense: 5′-ATGGACACCAAACATTTCCTGC<br>Antisense: 5′-GGGAGCTGATGACCGAGAAAG	62°C	HepG2 1/100<br>Caco-2 1/200<br>InEpC 1/50<br>RPTEC 1/200
SHP	Sense: 5′-GTGCCCAGCATACTCAAGAAG<br>Antisense: 5′-TGGGGTCTGTCTGGCAGTT	60°C	HepG2 1/1000<br>Caco-2 1/50<br>InEpC 1/25<br>RPTEC 1/50
SULT2A1	Sense: 5′-ACGGATTCGAGGCCACGTCC<br>Antisense: 5′-TCCGTTTCACTGAGTGCTGTA	62°C	HepG2 1/200<br>Caco-2 1/500<br>InEpC 1/50<br>RPTEC 1/200
TGR5	Sense: 5′-GACTTTGGACCATGAAGACCAG<br>Antisense: 5′-GCCCAGACGGAAGTTTCTTATT	60°C	HepG2 1/50<br>Caco-2 1/50<br>InEpC 1/25<br>RPTEC 1/200

SUPPLEMENTARY FIGURE LEGENDS

Supplementary Figure 1. Dose-dependent and gene-specific modulation of the bile acid-related transcriptome in human hepatoma HepG2 cells treated with EPA and DHA.

HepG2 cells were treated with DMSO (vehicle, 0.1% v/v) or DHA or EPA for 24H at 5, 15, 25 and 50 µM for mRNA measurements. Total RNA was extracted using the TriReagent® protocol to further measure mRNA levels by quantitative real time PCR as detailed in the materials and methods section.

Each data point represents the mean±S.D. of triplicate experiments. The results are representative of two independent experiments. Statistically significant differences were analyzed using one-way analysis of variance (ANOVA) (* $p < 0.05$).

Supplementary Figure 2. Time-dependent and gene-specific modulation of the bile acid-related transcriptome in human HepG2 cells treated with EPA and DHA.

HepG2 cells were treated with DMSO (vehicle, 0.1% v/v) or DHA or EPA at 50 µM for 6, 12, 18, 24 and 48H for mRNA measurements. Total RNA was extracted using the TriReagent® protocol to further measure mRNA levels by quantitative real time PCR as detailed in the materials and methods section.

Each data point represents the mean±S.D. of triplicate experiments. The results are representative of two independent experiments. Statistically significant differences were analyzed using one-way analysis of variance (ANOVA) (* $p < 0.05$).

Supplementary Figure 3. Additive and/or synergistic effects of the EPA+DHA combination on the bile acid-related transcriptome in human hepatoma HepG2 cells.

HepG2 cells were treated with DMSO (vehicle, 0.1% v/v) or DHA and/or EPA for 24H at 25 and/or 50 µM for mRNA measurements. Total RNA was extracted using the TriReagent® protocol to further measure mRNA levels by quantitative real time PCR as detailed in the materials and methods section.

Each data point represents the mean±S.D. of triplicate experiments. The results are representative of two independent experiments. Statistically significant differences were analyzed using one-way analysis of variance (ANOVA) (* $p < 0.05$).

Supplementary Figure 4. Preparation of the PUFA solution has minimum impact on the response of BA-related genes in HepG2 cells.

HepG2 cells were exposed to EPA/DHA (25/25 or 50/50µM) prepared either in DMSO or in culture medium containing 125µM BSA for 24H. Total RNA was extracted using the TriReagent® protocol and CYP7A1, CYP27 and MRP3 mRNA levels were determined by quantitative real time PCR as detailed in the materials and methods section.

Statistically significant differences were analyzed using one-way analysis of variance (ANOVA) (* $p < 0.05$).

Supplementary Figure 5. Time-dependent effects of n-3 PUFAs on the bile acid-induced activation of the pro-apoptotic Caspase 3 pathway in human hepatoma HepG2 cells.

HepG2 cells pretreated with DMSO (vehicle, 0.1% v/v) and DHA/EPA (50/50µM) **(A)**: for 24H and exposed to 100 µM BAs (CA, CDCA, LCA, CDA) for 0,5, 1, 2, 3, 6 and 24H, **(B)**: for 3, 6, 16 and 24H and exposed to 100 µM BA (CA, CDCA, LCA, CDA) for 2H. The caspase-3 activity was determined as indicated in the materials and methods section.

The results (mean±S.D.) are representative of two independent experiments. Statistical differences between two groups were analyzed using unpaired two-side *t*-test *versus* vehicle (*) or BAs (‡) ($p < 0.05$).

Supplementary Figure 6. Dose-dependent and additive/synergistic effects of EPA and/or DHA on the bile acid-related transcriptome in human colon carcinoma Caco-2 cells.

Human colon carcinoma Caco-2 were treated with DMSO (vehicle, 0.1% v/v) or DHA and/or EPA for 24H at 10, 25 and/or 50µM for mRNA measurements. Total RNA was extracted using the TriReagent® protocol to further measure mRNA levels by quantitative real time PCR as detailed in the materials and methods section.

Each data point represents the mean±S.D. of triplicate experiments. The results are representative of two independent experiments. Statistically significant differences were analyzed using one-way analysis of variance (ANOVA) (* $p < 0.05$).

Supplementary Figure 7. Time-dependent and gene-specific modulation of the bile acid-related transcriptome in colon carcinoma Caco-2 cells treated with EPA and DHA.

Human colon carcinoma Caco-2 were treated with DMSO (vehicle, 0.1% v/v) or DHA and EPA at 50 and 25μM respectively for 6, 12, 18, 24 and 48H for mRNA measurements. Total RNA was extracted using the TriReagent® protocol to further measure mRNA levels by quantitative real time PCR as detailed in the materials and methods section.

Each data point represents the mean±S.D. of triplicate experiments. The results are representative of two independent experiments. Statistical differences between two groups were analyzed using unpaired two-side *t*-test ($p < 0.05$).

Supplementary Figure 8. Time-dependent and gene-specific modulation of the bile acid-related transcriptome in RPTEC treated with EPA and DHA.

Human renal proximal tubule epithelial cells (RPTEC) were treated with DMSO (vehicle, 0.1% v/v) or DHA and EPA at 25 and 50μM, respectively for 6, 12, 18, 24 and 48H for mRNA measurements. Total RNA was extracted using the TriReagent® protocol to further measure mRNA levels by quantitative real time PCR as detailed in the materials and methods section.

Each data point represents the mean±S.D. of triplicate experiments. The results are representative of two independent experiments. Statistical differences between two groups were analyzed using unpaired two-side *t*-test ($p < 0.05$).

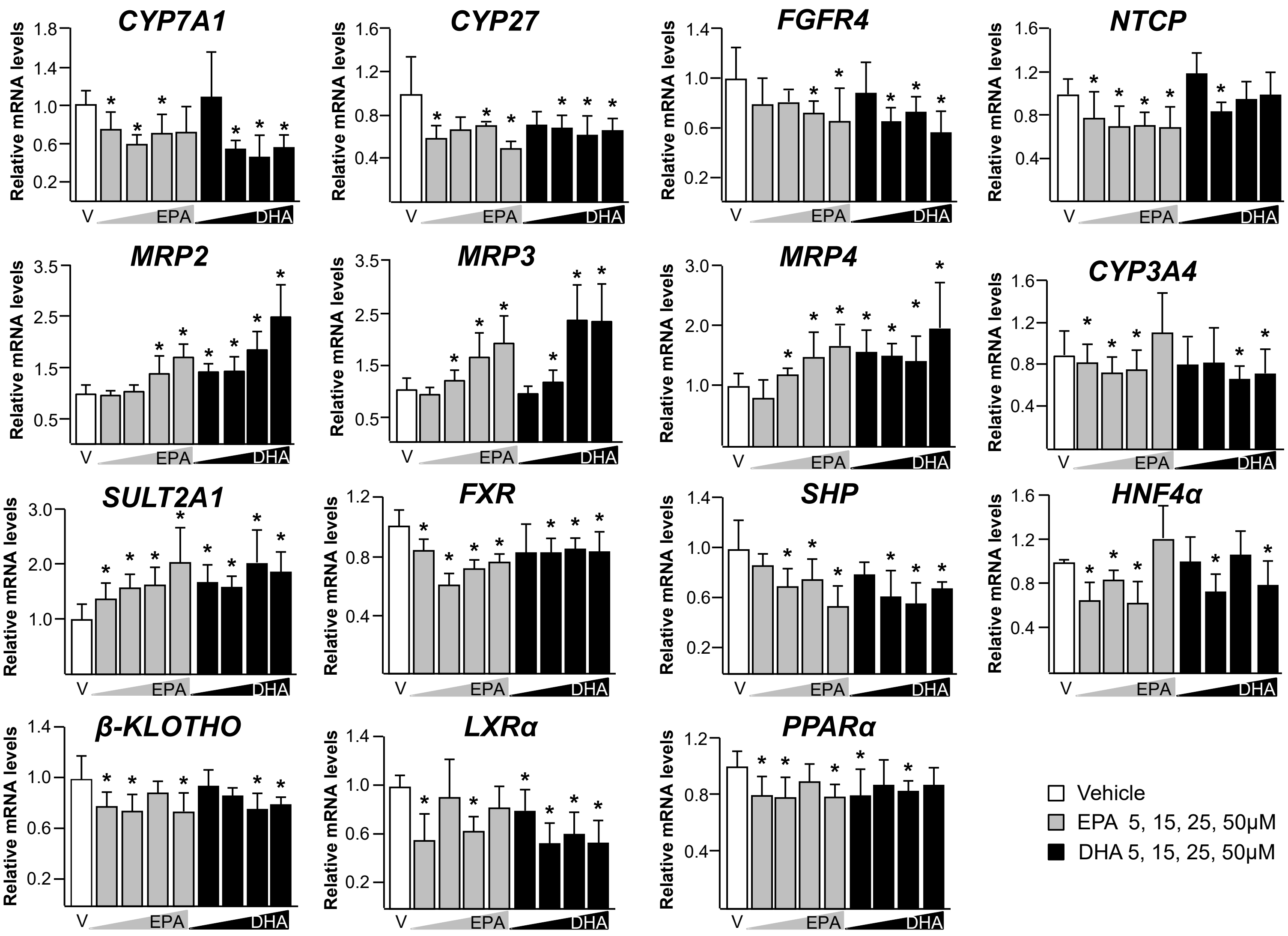

Supplementary Figure 1

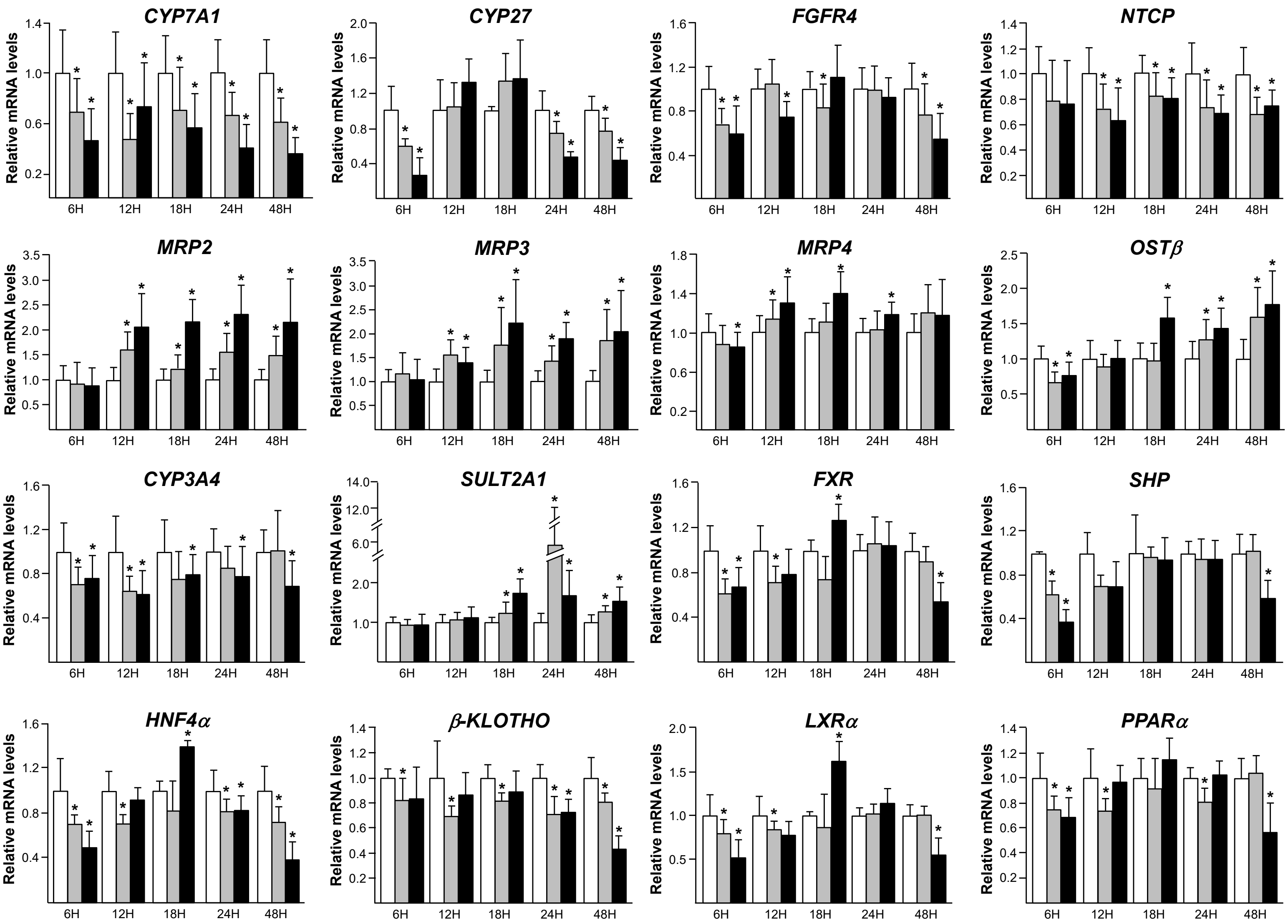

Supplementary Figure 2

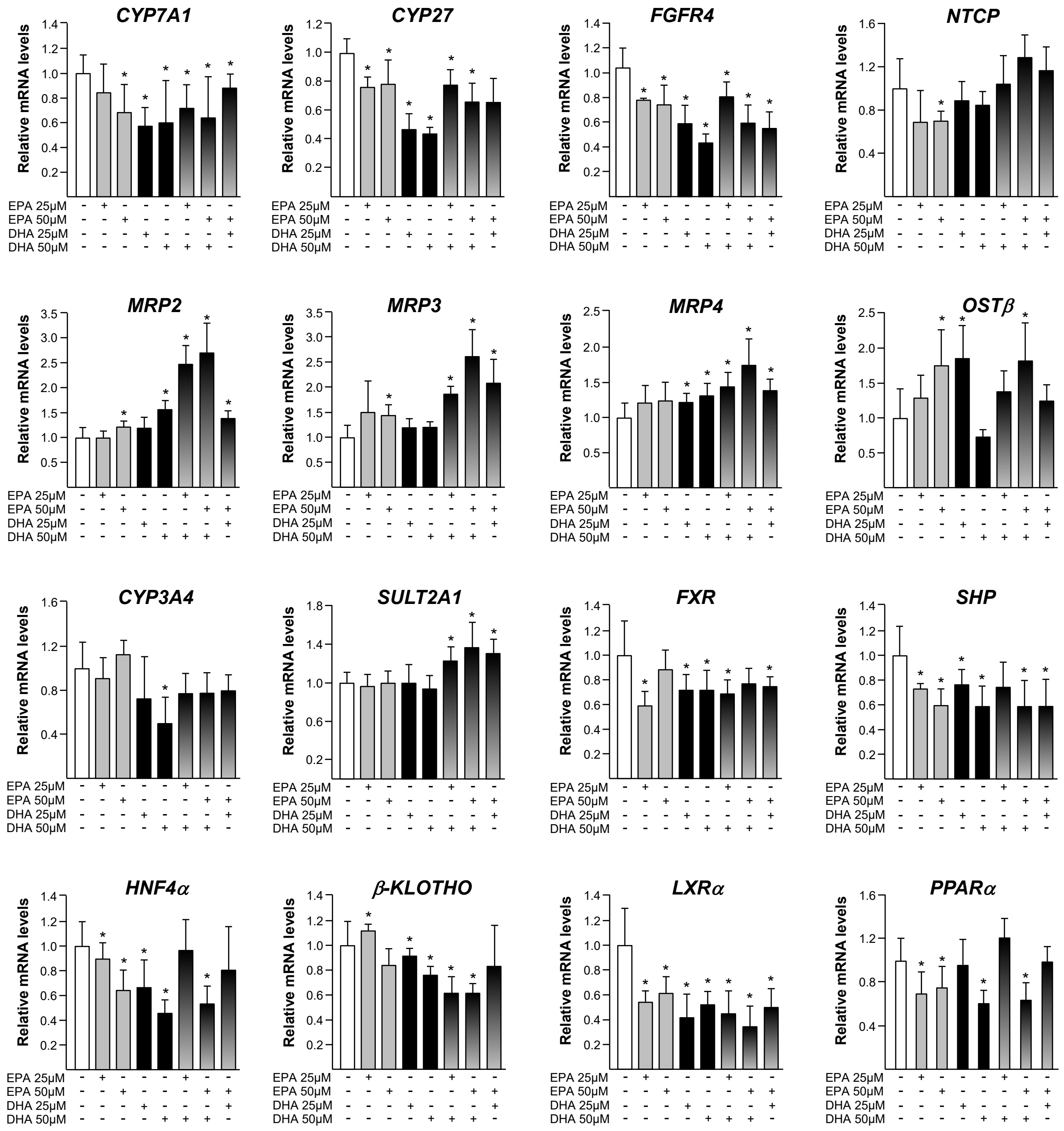

Supplementary Figure 3

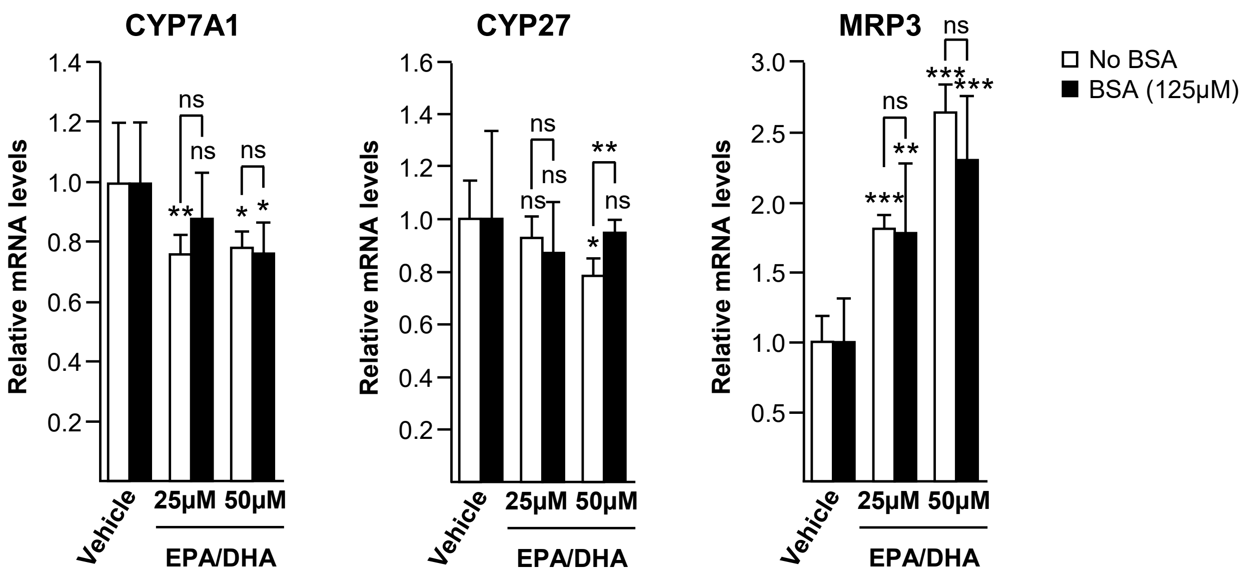

Supplementary Figure 4

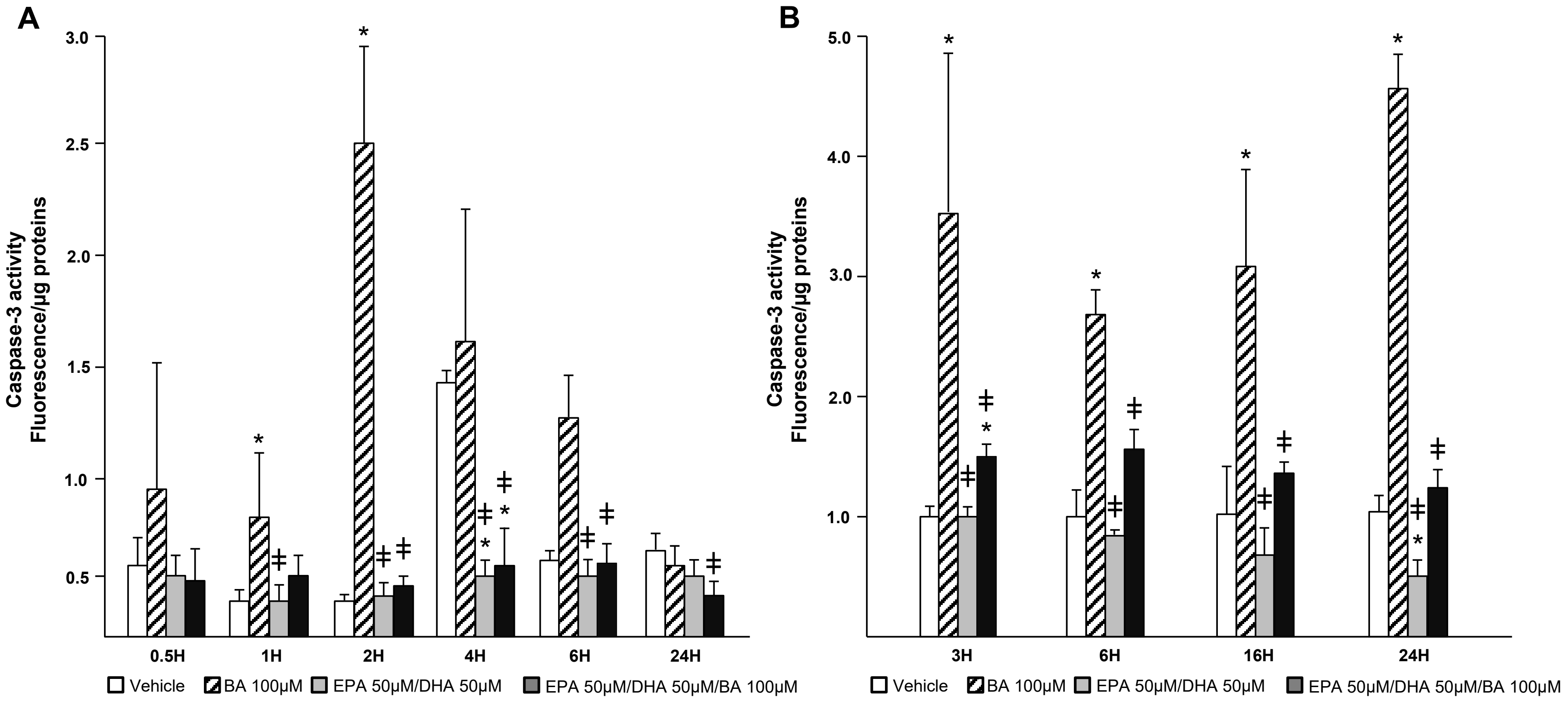

Supplementary Figure 5

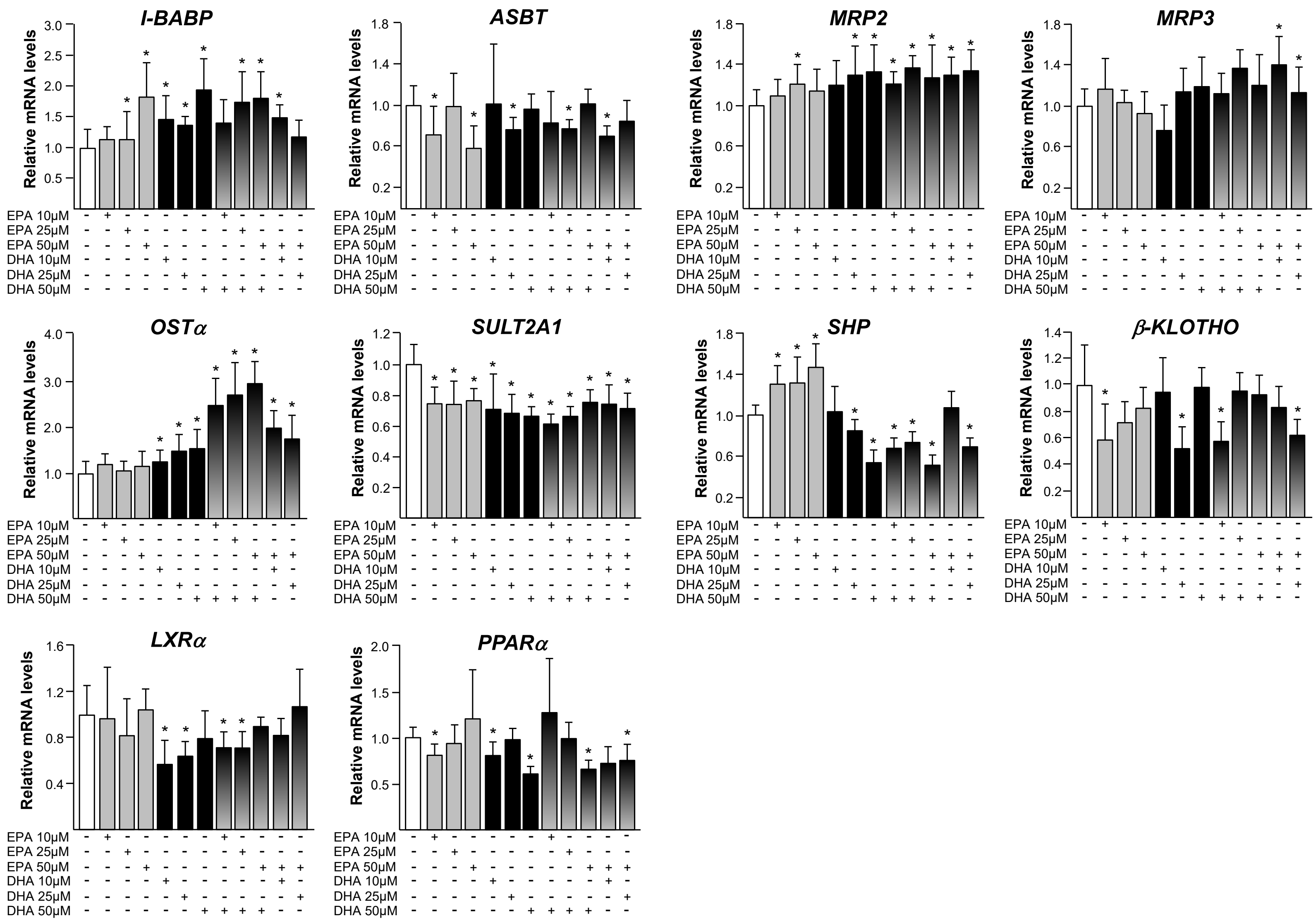

Supplementary Figure 6

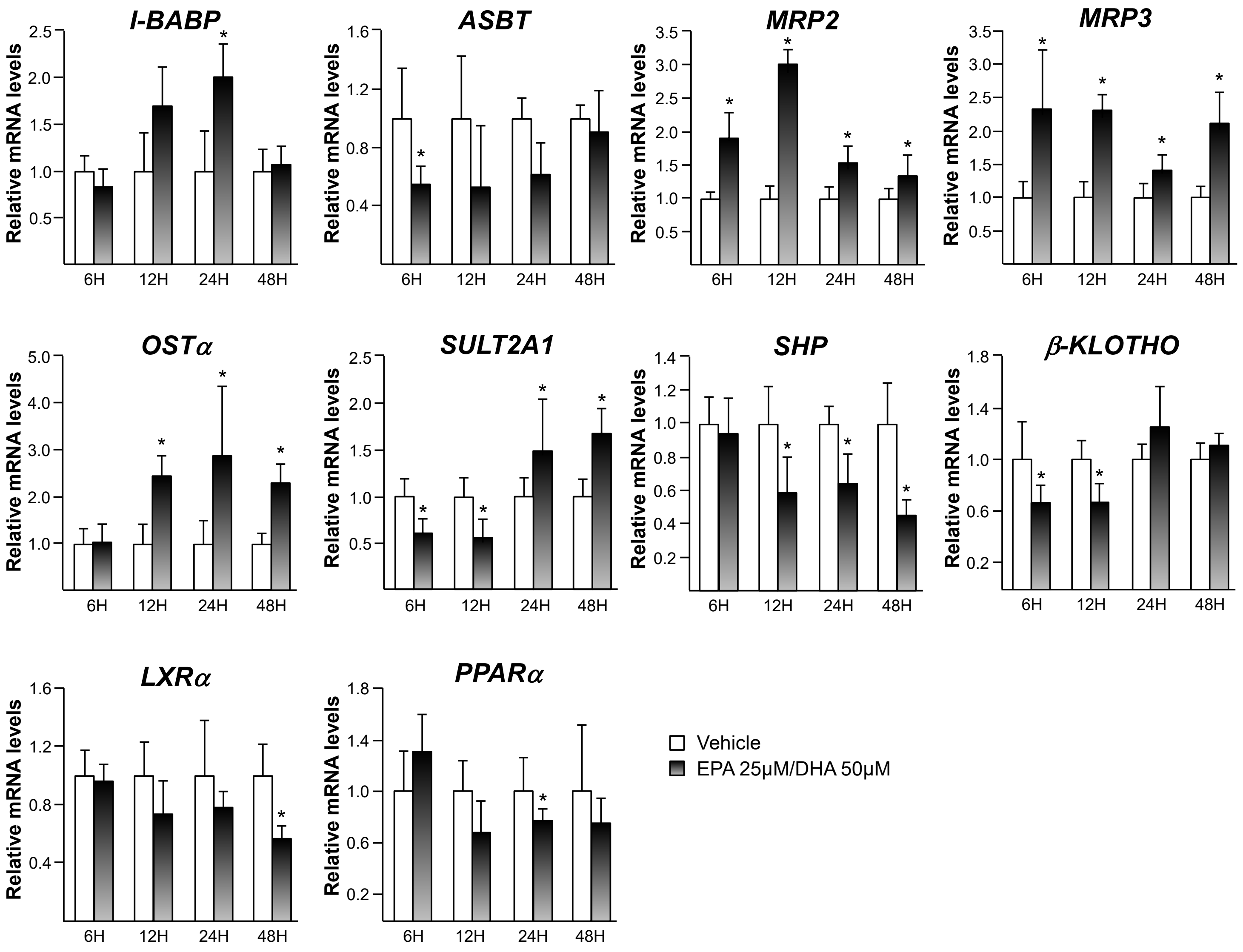

Supplementary Figure 7

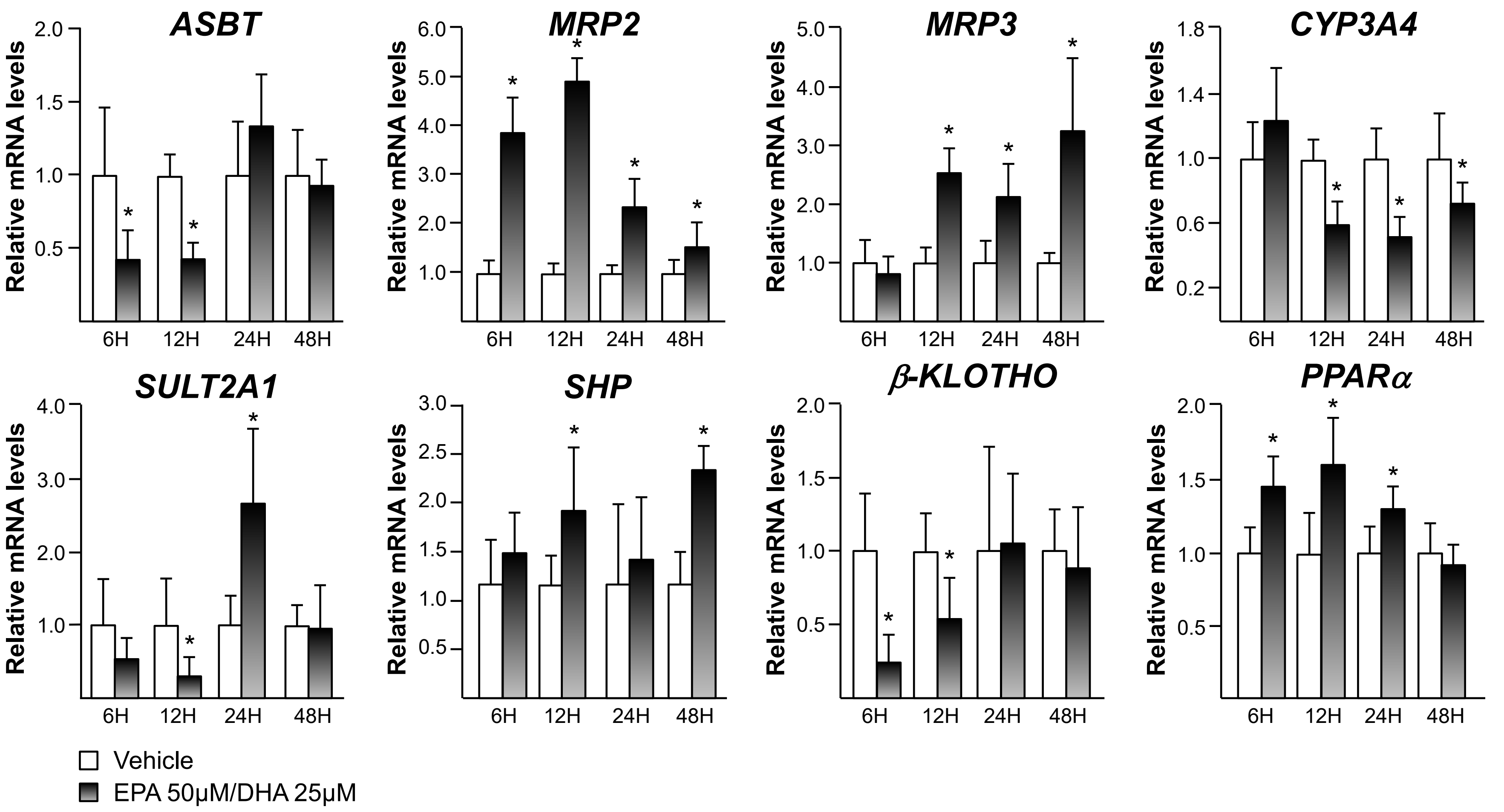

Supplementary Figure 8
